# Supplementary material for: Complexome profiling of the Chlamydomonas psb28 mutant reveals TEF5 as an early PSII assembly factor
Source: Plant Cell. 2025 Mar 17;37(6):koaf055. doi: 10.1093/plcell/koaf055 (PMC12164590; doi:10.1093/plcell/koaf055)
Supplement: koaf055_Supplementary_Data [file koaf055_supplementary_data.zip › psb28_interactive.zip!/plots/intensity/qualityPlots/peptides/CGL30_Cre03.g198950.t1.2.html]

### Cre03.g198950.t1.2

Trivial name: CGL30  
SeqCoverage [median]: 31.70  
Mass: 28.84 kDa  
  
 

| Sequence | MissedCleavage | Mass [Da] | wtIntensity | muIntensity | Score |
| --- | --- | --- | --- | --- | --- |
| EQGDVAVVVAPVLR | 0 | 1450.81 | 2640.25 | 5622.34 | 161.99 |
| EVGPPQR | 0 | 781.41 | 323.72 | 783.74 | 111.69 |
| FMDVGFNAK | 0 | 1027.48 | 0.00 | 1925.84 | 88.82 |
| NHYLVSATATK | 0 | 1203.62 | 0.00 | 1828.87 | 99.75 |
| NRVFILTVTSNAR | 1 | 1489.84 | 531.33 | 0.00 | 41.64 |
| TKEPDAEGFYTYTRPEGK | 2 | 2087.98 | 0.00 | 3185.13 | 55.33 |
| VFILTVTSNAR | 0 | 1219.69 | 0.00 | 5857.60 | 114.50 |
| VIQQSFR | 0 | 876.48 | 1617.59 | 6842.93 | 119.57 |
| VTLKEVGPPQR | 1 | 1222.70 | 646.52 | 2787.35 | 78.69 |
|  |  |  | 5759.4 | 28833.8 |  |
